# *Inonotus obliquus* upregulates muscle regeneration and augments function through muscle oxidative metabolism

**DOI:** 10.7150/ijbs.84970

**Published:** 2023-09-11

**Authors:** Chang-Lim You, Sang-Jin Lee, Jinwoo Lee, Tuan Anh Vuong, Hye-Young Lee, Se Yun Jeong, Akida Alishir, Allison S. Walker, Gyu-Un Bae, Ki Hyun Kim, Jong-Sun Kang

**Affiliations:** 1 Department of Molecular Cell Biology, Sungkyunkwan University School of Medicine, Suwon 16419, Republic of Korea.; 2 Research Institute of Aging Related Disease, AniMusCure Inc., Suwon 16419, Republic of Korea.; 3 School of Pharmacy, Sungkyunkwan University, Suwon 16419, Republic of Korea.; 4 Department of Chemistry, Vanderbilt University, Nashville, TN 37235, United States.; 5 Department of Biological Sciences, Vanderbilt University, Nashville, TN 37235, United States.; 6 Drug Information Research Institute, Muscle Physiome Research Center, College of Pharmacy, Sookmyung Women's University, Seoul 04310, Republic of Korea.

**Keywords:** *Inonotus obliquus*, PGC-1α, Muscle regeneration, Muscle atrophy, Muscle oxidative metabolism

## Abstract

Skeletal muscle wasting related to aging or pathological conditions is critically associated with the increased incidence and prevalence of secondary diseases including cardiovascular diseases, metabolic syndromes, and chronic inflammations. Much effort is made to develop agents to enhance muscle metabolism and function. *Inonotus obliquus* (*I*. *obliquus*; IO) is a mushroom popularly called chaga and has been widely employed as a folk medicine for inflammation, cardiovascular diseases, diabetes, and cancer in Eastern Europe and Asia. However, its effect on muscle health has not been explored. Here, we aimed to investigate the beneficial effect of IO extract in muscle regeneration and metabolism. The treatment of IO in C2C12 myoblasts led to increased myogenic differentiation and alleviation of dexamethasone-induced myotube atrophy. Network pharmacological analysis using the identified specific chemical constituents of IO extracts predicted protein kinase B (AKT)-dependent mechanisms to promote myogenesis and muscle regeneration. Consistently, IO treatment resulted in the activation of AKT, which suppressed muscle-specific ubiquitin E3 ligases induced by dexamethasone. IO treatment in mice improved the regeneration of cardiotoxin-injured muscles accompanied by elevated proliferation and differentiation of muscle stem cells. Furthermore, it elevated the mitochondrial content and muscle oxidative metabolism accompanied by the induction of peroxisome proliferator-activated receptor γ coactivator α (PGC-1α). Our current data suggest that IO is a promising natural agent in enhancing muscle regenerative capacity and oxidative metabolism thereby preventing muscle wasting.

## Introduction

*Inonotus obliquus* (*I. obliquus*) is a mushroom belonging to the family Hymenochaetaceae (Basidiomycota) popularly called chaga in Russian folk medicine 1. Chaga mushroom is a parasitic fungus that grows on birch in the forests of Northern European countries 2. The mushroom is widely consumed as tea, syrup, bath agents, or concentrate 1,3 and has been widely employed as a folk medicine for inflammation, cardiovascular diseases, diabetes, and cancer in Eastern Europe and Asia 1,4,5. The main bioactive compounds of *I. obliquus* are polysaccharides, polyphenols, melanin, and triterpenes 6. By using whole *I. obliquus* extract or its components, studies have shown antitumor, anti-inflammatory, immunomodulatory, antioxidant, hypoglycemic, and hypolipidemic activity of *I. obliquus*. However, its potential effect on myogenesis and muscle regeneration has not been investigated so far.

Decline in skeletal muscle mass and function is a serious public health issue without effective cure. It not only severely compromises body movement and daily activities, but also increases the incidence and prevalence of secondary diseases leading to frailty and reduced healthy life span 7. The age-associated decline in muscle mass and strength, which is referred to as sarcopenia, is attributed to reduced regenerative capacity of the muscle, declined mitochondrial function, and dysregulated immune responses 8,9. Among diverse strategies, so far exercise seems to be most effective measure to improve muscle function in sarcopenic patients so far 10,11. Thus, much efforts have been made to elucidate the underlying molecular mechanisms of the exercise effect on muscle mass and function. Peroxisome proliferator-activated receptor γ coactivator-1 alpha (PGC-1α) is a key regulator exerting exercise effect by eliciting gene expression related to mitochondrial biogenesis, fatty acid oxidation, angiogenesis, suppression of muscle atrophy and muscle regenerative capacity 12. Mice lacking PGC-1α display impaired oxidative muscle metabolism and exercise capacity 13,14. Thus, elevation of PGC-1α seems to be an attractive strategy to intervene muscle atrophy and weakness related to aging. Indeed, several studies have reported the beneficial effect of PGC-1α increase in aging related metabolic diseases and muscle wasting 15,16. Two PGC-1α inducers, AICAR (AMPK agonist) and metformin (anti-diabetes) have shown to replicate the effect of exercise on muscle function 17-19. Protein kinase B (AKT) signaling is another promyogenic signaling pathway critical for cell survival and differentiation of myoblasts triggered by the external signals like insulin-like growth factor 20-23. AKT can activate the action of myogenic regulatory factors like MyoD and MEF2 thereby inducing muscle specific gene expression contributing to myogenesis and muscle regeneration 22,24. AKT signaling also plays a key role in anabolic responses associated with increased protein synthesis and resulting in muscle growth 23,25.

The wide range of biological activity of *I. obliquus* in cells to modulate oxidative stress, immune response, and metabolic properties make the natural resource an attractive candidate to promote muscle health. Therefore, we investigated the effect of *I. obliquus* extract on myogenesis and myotube atrophy models of C2C12 myoblasts and muscle regeneration model of mice. We show that *I. obliquus* treatment improves myoblast differentiation and prevents myotube atrophy induced by dexamethasone (DEX). In addition, *I. obliquus* enhances muscle stem cell proliferation and regeneration post muscle injury. Further analysis revealed an increase in mitochondrial content and oxidative muscle metabolism in *I. obliquus*-treated muscles. Finally, we present the activation of AKT and PGC-1α expression as the potential underlying mechanism of *I. obliquus*'s biological activities in muscle.

## Materials and methods

### Chemicals and Reagents

Wild-type C57BL/6 male mice were purchased from (Orient-Bio, Seongnam, Korea). Fetal bovine serum (FBS), horse serum (HS) and Dulbecco modified Eagle's medium (DMEM) were purchased from Thermo Scientific (Waltham, MA). 3-(4,5-dimethylthiazol-2-yl)-2,5-diphenyltetrazolium bromide (MTT), DEX and all other chemicals were from Sigma-Aldrich (St. Louis, MO). Antibodies were purchased as following: Myosin heavy chain (MHC, Developmental Studies Hybridoma Bank (DSHB), Iowa, IA), MyoD (Novus biologicals, Littleton, CO), Myogenin, Myoglobin, total-OxPHOS (Abcam, Cambridge, MA), total AKT, phospho-AKT, total p38, phospho-p38, β-actin (Cell Signaling Technology, Beverly, MA), MuRF1, Atrogin-1, HSP90 (Santa Cruz Biotechnology, Santa Cruz, CA), PGC-1α (Calbiochem, San Diego, CA) and β-tubulin (Zymed, South San Francisco, CA).

### Plant material

The fruiting bodies of chaga mushroom (*I. obliquus*) were purchased at Kyungdong herbal market, Seoul, Korea, in July 2019, and were identified by one of the authors (K. H. Kim). A voucher specimen (SKKU CG-2019-07) has been deposited in the herbarium of the School of Pharmacy, Sungkyunkwan University, Suwon, Korea.

### Extraction procedure of IO1 and IO2

Dried fruiting bodies of *I. obliquus* (500 g) were dried, chopped, and the chopped material was extracted using distilled water (3 × 500 mL × 5 h) under reflux, and then filtered. The filtrate was evaporated under a vacuum to obtain an aqueous extract (20.2 g) of *I. obliquus* (IO1). Dried fruiting bodies of *I. obliquus* (500 g) were partially chopped and extracted with 70% EtOH (3 × 500 mL) for 2 days at room temperature and filtered. The filtrate was concentrated under vacuum pressure, generating a crude EtOH extract (22.5 g) of *I. obliquus* (IO2). The IO1 and IO2 extracts were prepared as stock solutions at 1.0 μg/mL in dimethyl sulfoxide (DMSO, Sigma-Aldrich). All stock solutions were aliquoted and stored at -80 °C until use.

### UPLC Conditions

The samples were analyzed on an Agilent 1290 Infinity II UPLC coupled to a G6545B Q-TOF MS system with dual ESI source (Agilent Technologies, USA). All samples were separated on an Agilent ZORBAX RRHD Eclipse Plus C_18_ column (50 × 2.1 mm, 1.8 µm) using 0.1% formic acid-deionized water (A) and acetonitrile (B). The optimized gradient elution program was as follows: 0-10 min, 10-100% B; 10-12 min, 100% B; 12-15 min, 10% B. The temperature was set at 20 °C, and the injection volume was 1 µL. The spectral acquisition rate and time were set at 1 spectra/s and 1000 ms/spectrum each. The flow rate was 0.3 mL/min. The wavelength was set at 210 nm. The concentration of samples (distilled water extract and EtOH extract) was prepared as 1000 ppm and injected 3 times in cases of positive ion-mode and negative ion-mode respectively.

### ESI Q-TOF MS Analysis

The Agilent Q-TOF G6545B mass spectrometer (Agilent Technologies) was operated in positive-ion and negative-ion mode. The parameters of the ESI source were optimized as follows: gas temperature 320 °C, gas flow 8 L/min, nebulizer pressure 35 psi, sheath gas temperature 350 °C, sheath gas flow 11 L/min, capillary voltage 3500 V, nozzle voltage 1000 V, and fragmentor voltage 100 V. Internal references (Purine and HP-0921) were adopted to modify the measured masses in real time. The reference masses in positive ion mode were at *m/z* 121.0508 and 922.0097. The reference masses in negative ion mode were at *m/z* 119.0363 and 1033.9881. The full scan range of the mass spectrometer was *m/z* 100-1700 for MS.

### Data processing

Data processing process was conducted in cases of positive-ion mode and negative-ion mode respectively. The obtained UPLC Q-TOF MS raw data were further processed by Agilent MassHunter Profinder software (version 10.0, Agilent, America). The batch recursive feature extraction (small molecules/peptide) algorithm was applied to extract compounds from the total ion chromatograms (TICs) according to their molecular features including *m/z*, retention time and ion intensities, and the main parameters of MFE were optimized. Also, this algorithm used to bind and align compounds within the batch by retention time and mass tolerance; the tolerance windows of retention time and accurate mass were 0.3 min and 20 ppm, respectively. The restrict retention time range and *m/z* range were set at 0.5-13.0 min and 100 to 1700 *m/z* respectively. Low-abundance ions can be hard to identify if the precursor ion intensity is low, generally below 1000 counts for an Agilent Q-TOF. To produce a matrix containing fewer biased and redundant data, the thresholds of peak filters was set at 1000 counts. Missing peaks were filtered according to their frequency, and metabolites that appeared in 100% of samples in at least one group were retained. All the extracted compounds were output to create a .pfa (Profinder Archive) file, which can be imported into Mass Profiler Professional (MPP) software (version 15.1, Agilent) for further data analysis. Normalization (percentile shift), defining the sample sets, baselining (median of all samples), filtering by frequency, and significance analysis (T-test; *p*-value cut-off: 0.05; fold change cut-off: 2.0) were applied to process the data. The generated data were then processed for principal component analysis (PCA) by MPP software (version 15.1, Agilent). The successfully obtained specific metabolites (Table S1) for IO1 were identified by their accurate mass-to-charge ratio (*m/z*) values and the MS/MS fragmentation ion for each of the corresponding accurate *m/z* values aided by CFM-ID 4.0, a software tool for MS/MS spectral prediction and MS-based compound identification at http://cfmid.wishartlab.com as well as literature survey of *I. obliquus* compounds reported and comparison to authentic standards.

### Network pharmacology analysis

Predicted protein targets of metabolites were identified using the STITCH 26, SwissTargetPrediction 27, and ChEMBL 28 databases. We included a target in the network if it was a human protein and had a greater than 90% probability in SwissTarget or was predicted to be active at 90% confidence in ChEMBL or if a connection was present between the molecule and the target in the STITCH network. Under these criteria, there were no hits from SwissTarget or STITCH, but there were hits from ChEMBL. A network of these compounds and targets was built using STITCH and a network of the protein-protein interactions was built using STRING 29. In the STRING network, we observed that the following gene ontologies relevant to muscular function were enriched: skeletal muscle tissue growth, neuromuscular synaptic transmission, skeletal muscle contraction, muscle contraction mainly due to the presence of acetylcholine receptor subunits in the network. Important target nodes were identified by comparing the degree, betweenness centrality, and closeness centrality to the median values. Betweenness and closeness centrality were calculated using the Networkx python package 30.

### Animal experimental design

The wild-type C57Bl/6 male mice were obtained from Orient-Bio (Seongnam, Korea) and maintained until sacrificed. All mice were maintained at 23 ℃ with a 12:12 h light-dark cycle and free access to food and water. The mice were orally administrated a daily dose of 4 mg/kg IO for 4 weeks (4-month-old mice, young) and control mice were administrated the same amount of vehicle drinking water. For the muscle atrophy experiment, the 4-month-old mice were orally administrated either vehicle or IO for 1 week prior to injecting cardiotoxin (CTX) and were administrated until be sacrificed. Then, they were victimized on Day 3 and Day 21 after injection. All animals were sacrificed after fasting for 16 h with ad libitum to water, and their muscles were harvested 4 h after the last administration of IO. All animal experiments were approved by the Institutional Animal Care and Research Advisory Committee at Sungkyunkwan University school of Medicine (SUSM) and complied with the regulations of the institutional ethics committee.

### Cell culture and cell viability assay

C2C12 myoblasts were cultured as previously described 31. They were grown in DMEM (Dulbecco's Modified Eagle Medium high glucose; Thermo Scientific, Waltham, MA) containing 15% FBS (growth medium, GM), 10 units/mL penicillin and 10 µg/mL streptomycin (Welgene, Daegu, Korea) at 37 ℃, 5% CO_2_. To induce differentiation of C2C12 myoblasts, cells at near confluence were changed growth medium into DMEM containing 2% HS (differentiation medium, DM) and myotube formation was observed at 2 or 3 days after differentiation. For the DEX-induced atrophy study, C2C12 cells were induced to differentiate in differentiation medium for 3 days and treated with 100 µM DEX and indicated concentration of IO, followed by incubation in differentiation medium for additional 1 day 32.

Cell viability assay was quantified using MTT colorimetric assay. In briefly, C2C12 cells were seeded in a 96-well plate (5 × 10^4^ cells/well) overnight and treated with the indicated concentration of IO for 24 h. MTT solution was added to the each well, and the cells were incubated for 4 h at 37 ℃. The optical density was measured at 540 nm.

### Western blotting and immunostaining

Western blot analysis was performed as previously described 33. Briefly, cells were lysed in cell extraction buffer (10 mM Tris-HCl, pH 8.0, 150 mM NaCl, 1 mM EDTA, 1% Triton X-100) containing complete protease inhibitor cocktail (Roche Diagnostics, Basel, Switzerland), followed by SDS-PAGE and incubation with primary and secondary antibodies.

Immunostaining for MHC expression was carried out as previously described 33. Briefly, the differentiated cultures were then immunostained for MHC antibodies and Alexa 568- or 488-conjugated secondary antibodies (Molecular Probes, Eugene, OR). Images were captured and processed with a Nikon ECLIPSE TE-2000U microscope and NIS-Elements F software (Nikon, Tokyo, Japan). To analyze the efficiency of myotube formation, the MHC-positive myotubes containing two to nine, or ten or more nuclei, were quantified at least three times and measured using IMAGE J software (version 1.53e; National Institutes of Health, Bethesda, MD). MHC-positive myotubes with 10 and more nuclei were measured transverse diameter in six fields. Quantification of myotube diameter was performed with IMAGE J software. Average myotube diameter is presented as means determination of six fields ± 1 standard deviation (SD).

### RNA isolation and quantitative RT-PCR

Total RNA extraction and quantitative RT-PCR analysis was performed as previously described 31. Tissues were homogenized by FastPrepR-24 (MP Biomedicals, Santa Ana, CA) and extracted using the easy-spin Total RNA Extract kit (iNtRON, Seongnam, Korea). Gene expression fold change was normalized against the expression of 18S ribosomal RNA. The sequences of the primers used in this study are provided in Table 1.

### Cryosections, staining analysis, and fiber size measurement

Muscle tissue was embedded in Tissue-Tek OCT Compound (Sakura Finetek, Nagano, Japan), and 7mm thick serial sections for staining were cut using a cryomicrotome. To analyze the NADH dehydrogenase activity, we dried the sectioned tissues for 10 min in room temperature and incubated in 0.9 mM NADH and 1.5 mM nitro blue tetrazolium (NBT; Sigma-Aldrich) in 3.5 mM phosphate buffer (pH 7.4) for 30 min. To analyze the succinate dehydrogenase (SDH) activity, we incubated the sections for 1 h in 50 µM sodium succinate and 0.3 mM nitro blue tetrazolium in 114 mM phosphate buffer containing K-EGTA (Sigma-Aldrich). Myh immunostaining of muscle tissue sections was performed in the sequence of fixation, permeation, and incubation with primary antibodies against MyhIIa and MyhⅡb (DSHB) and laminin (Abcam). Images were captured and proceed with a Nikon ECLIPSE TE-2000U using NIS-Elements F software. Myofiber area was measured with ImageJ software. For muscle histology, the cryosections were stained with Mayer's hematoxylin and eosin (BBC Biomedical, McKinney, TX). The images were captured using a Nikon ECLIPS TE-2000U.

### PGC-1α Luciferase Assay

PGC-1α luciferase assay was performed as previously described 34. C2C12 cells were transfected with an expression plasmid for luciferase responsive to the 2 kb promoter region of PGC-1α (Addgene plasmid #8887, Addgene, Cambridge, MA, USA), PGC-1α promoter luciferase delta CRE site (Addgene plasmid #8888, Addgene) and PGC-1α promoter luciferase delta MEF site (Addgene plasmid #8889, Addgene) using Lipofectamine 2000 (Invitrogen) according to the manufacturer's instructions. After 24 h of transfection, the cells were incubated in differentiation media with IO1. The cells were lysed with Reporter Lysis Buffer (Promega), and luciferase assays were performed using a Luciferase Assay System kit and a luminometer (Berthold Technology, Bad Wildbad, Germany). The Transfection efficiency was normalized based on the co-transfected b-galactosidase enzyme activity measured using an assay kit (Promega). Experiments were performed in triplicates and repeated at least three times independently.

### Statistical analysis

Values are expressed as mean ± SD or ± standard error of mean (SEM), as indicated in the figure legends. Differences were considered statistically significant at or under values of *P* < 0.05.

## Results

### Effects of *Inonotus obliquus* extracts on myogenic differentiation of C2C12 myoblasts

**Figure 1:**
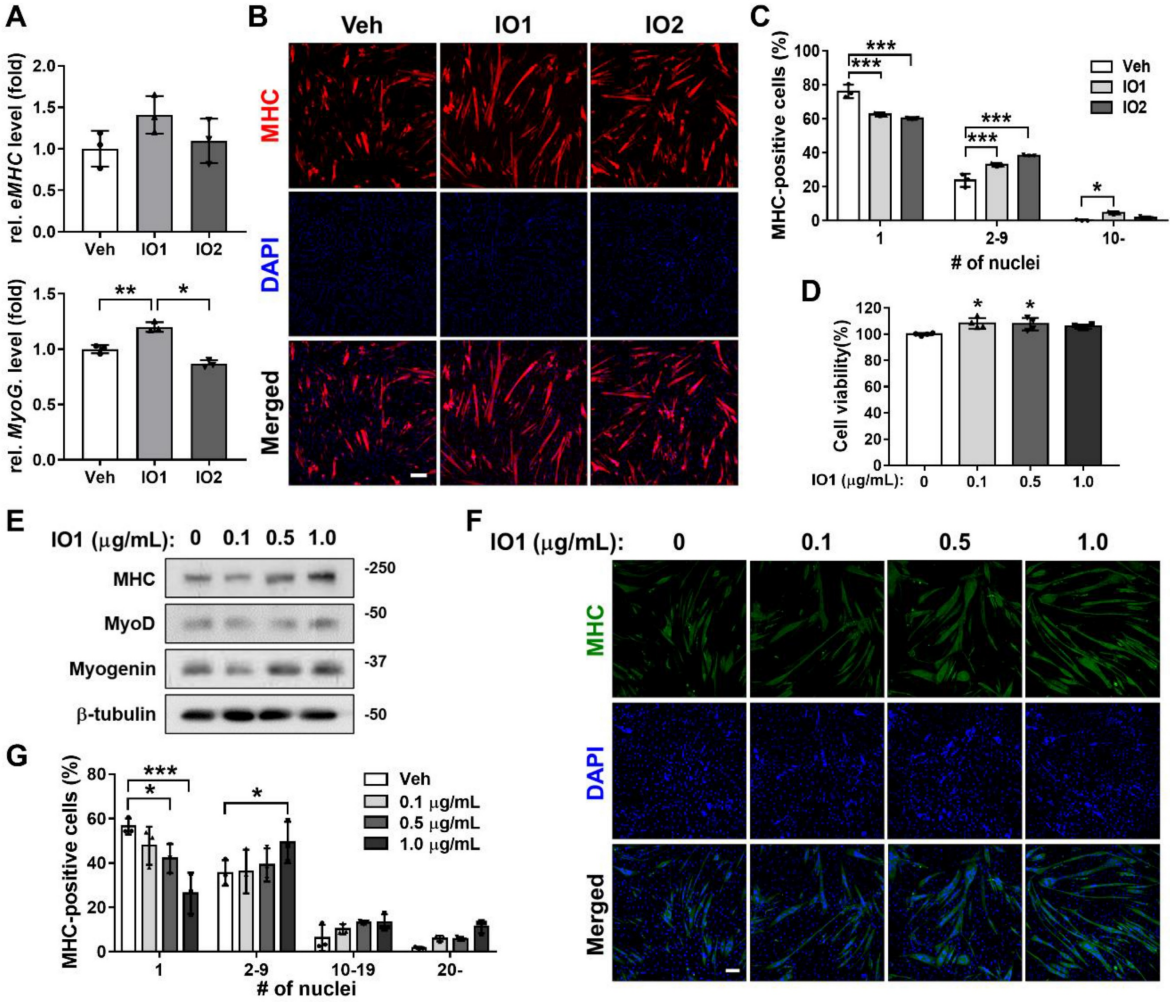
** Effect of IO on myogenic differentiation of C2C12 myoblast cells.** (A) Comparison of IO1 and IO2 in the degree of myoblast differentiation. Expression of eMHC and Myogenin in C2C12 cells treated with vehicle, IO1 (1.0 µg/mL), or IO2 (1.0 µg/mL) for 1 day in DM were analyzed by qRT-PCR. The data were normalized using 18s RNA, *n* = 3. eMHC and Myogenin expression levels were further normalized to the expression level of vehicle (DMSO). (B) Immunostaining for MHC expression in C2C12 cells treated with DM for 1 day and treated with vehicle, IO1 (1.0 µg/mL), or IO2 (1.0 µg/mL) for 1 day in DM. Scale bar, 50 µM. (C) Quantification of myotube formation from data shown in panel B, *n* = 3. (D) Cell viability of C2C12 cells in the presence of 0, 0.1, 0.5, and 1.0 µg/mL IO for 1 day, *n* = 4. (E) Immunoblot analysis of C2C12 cells treated with indicated concentration of IO and differentiated in DM for 2 days. Cell lysates were subjected to antibodies against MHC, MyoD, Myogenin, and β-tubulin. (F) Immunostaining of C2C12 cells treated with indicated concentration of IO for MHC expression (green). Scale bar, 50 µM. (G) Quantification of myotube formation from data shown in panel F, *n* = 3. Data are expressed as the means ± SD. Asterisks indicate significant difference from the control. *P < 0.01, **P < 0.05, and ***P < 0.001.

To investigate the effect of *Inonotus obliquus* extract (IO) on myogenic differentiation, we prepared two types of IO: IO1 (aqueous extract) and IO2 (ethanol extract). C2C12 myoblasts were induced to differentiate for 1.5 days in the presence of vehicle, IO1, or IO2 in DM and subjected to the assessment of myoblast differentiation by quantitative real-time polymerase chain reaction (qRT-PCR). IO1 treatment substantially elevated the expression of muscle-specific genes, embryonic myosin heavy chain (eMHC) and Myogenin, compared to vehicle or IO2 treatment (Figure 1A) and in a dose-dependent manner (Figure S1), while IO2 did not show much effect on the expression of muscle-specific genes. To further examine the promyogenic effects of IOs, myotube formation was assessed by MHC immunostaining. Both IO1 and IO2 treatment in C2C12 myoblasts promoted the formation of larger MHC-positive multinucleated myotubes compared to vehicle-treated cells (Figure 1B and C). In particular, IO1 treatment effectively elevated the proportion of larger MHC-positive myotubes containing ten or more nuclei. However, the effects of IO2 were lesser than IO1. Therefore, we decided to place more emphasis on the investigation of IO1 (hereafter designated as IO). Up to a concentration of 10 μg/mL, IO did not show any cytotoxicity, as assessed by the MTT assay (Figure 1D). However, significant cytotoxicity was observed with the treatment of IO starting at a concentration of 50 μg/mL (Figure S2). IO-treated C2C12 cells displayed incremental increase in the expression of MHC, MyoD, and Myogenin in a dose-dependent manner (Figure 1E), which was also confirmed by immunostaining of MHC (Figure 1F). For the quantification of myotube formation, the nuclei MHC-positive myotubes were counted. Treatment with IO significantly increased the proportion of larger myotubes with 20 or more nuclei, while mononucleated myocytes decreased in a concentration-dependent manner (Figure 1G). These data suggest that IO enhances C2C12 myoblast differentiation without overt cytotoxicity.

### Characterization of the chemical constituents in the active IO and network pharmacological analysis

**Figure 2:**
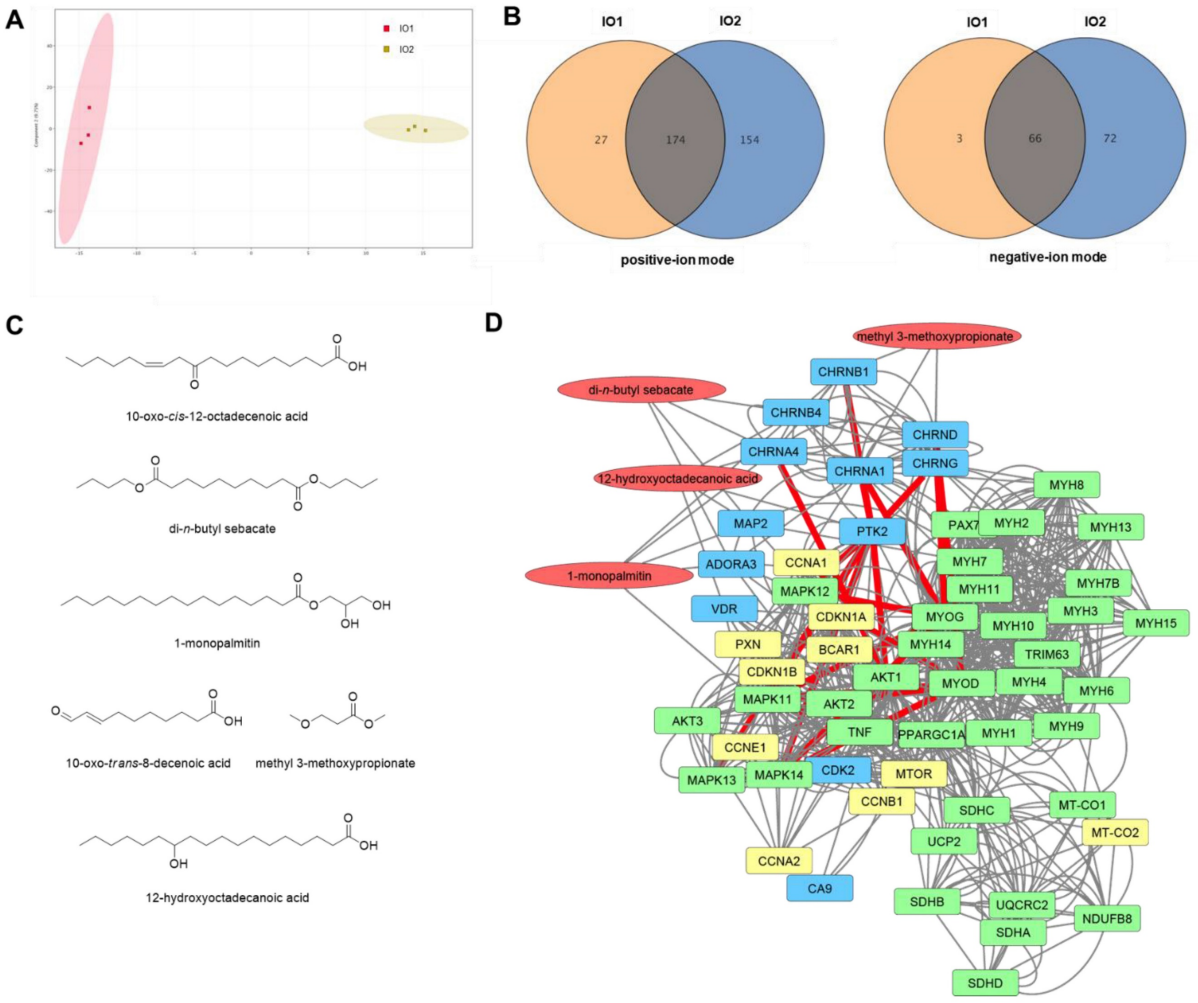
** Analysis of the chemical constituents in IO1 and network pharmacological analysis.** (A) Principal component analysis (PCA) of IO1 and IO2 samples based on the metabolomics analysis in negative-ion mode. (B) Venn diagrams of pairwise analyses (IO1 versus IO2). (C) The identified specific chemical constituents in IO1. (D) Pharmacological network of IO1. Pharmacological network was built using SwissTargetPrediction, ChEMBL, STRING, and STITCH. The specific chemical constituents identified from IO1 are shown in red, predicted targets in blue, proteins of interest in green, and proteins added to the network by STRING or STITCH in yellow. Edges hypothesized to be important in the mechanism of action based on node importance and literature search are highlighted in red.

To investigate the potential underlying mechanism of the effects of *I. obliquus* in muscle, the chemical constituents of *I. obliquus* extracts were analyzed by ultra-high-performance liquid chromatography (UPLC) coupled with quadrupole time-of-flight mass spectrometry (Q-TOF MS). Since IO1 showed greater enhancement of C2C12 myoblast differentiation than IO2, we presumed that the specific metabolites in the aqueous extract of *I. obliquus* (IO1) are the potential bioactive compounds. The obtained typical total ion chromatograms (TICs) for the two types of IO were similar (Figure S3) with most of the major peaks appearing in TICs of both IO1 and IO2. However, the principal component analysis (PCA) of the UPLC-Q-TOF MS data showed that the IO1 and IO2 samples were clearly separated (Figure 2A), indicating the different metabolite profiles for IO1 and IO2. In the positive-ion mode, there were 201 chemical constituents in IO1 and 328 chemical constituents in IO2. In the negative-ion mode, there were 69 chemical constituents in IO1 and 138 chemical constituents in IO2. To verify the specific metabolites in IO1, pairwise analysis of IO1 versus IO2 was performed using a Venn diagram (Figure 2B). The results indicated that 27 and 3 specific metabolites of IO1 were found in the positive-ion mode and negative-ion mode, respectively. The specific metabolites obtained were checked manually, since they contained redundant signals caused by different isotopes and in-source fragmentation, and the redundant signals were manually removed. Finally, the highly reproducible and non-redundant metabolite signals were taken as 6 specific chemical constituents in IO1 (Figure 2C).

Next, network pharmacology analysis was performed to explore the potential mechanisms of *I. obliquus* on myoblast differentiation and muscle regeneration using the identified specific chemical constituents of IO1. We used STITCH 26, SwissTargetPrediction 27, and ChEMBL 28 to identify possible protein targets of IO1. The ChEMBL database was the only tool that identified candidate target proteins with high confidence. We then constructed a network of the target proteins identified by ChEMBL and proteins of interest associated with skeletal muscle tissue growth, contraction, and neuromuscular synaptic transmission using STRING 29 and STITCH 26. In this network, the neuronal acetylcholine receptor was a predicted target of di-*n*-butyl sebacate, 1-monopalmitin, and 12-hydroxyoctadecanoic acid and the acetylcholine receptor was a predicted target of methyl 3-methoxypropionate (Figure 2D). The acetylcholine receptors were in turn linked to many of the proteins of interest including protein kinase B (AKT1), myoblast differentiation protein (MYOD), myogenin (MYOG), myosin heavy chain 2, 3, 8 (MYH2, MYH3, MYH8), E3 ubiquitin-protein ligase (TRIM63), and peroxisome proliferator-activated receptor g coactivator (Pgc-1α). In addition, 12-hydroxyoctadecanoic acid was predicted to interact with focal adhesion kinase 1 (PTK2), which was identified to be a major node that links between the acetylcholine receptors and downstream kinases such as AKT and MAP in the network. Accordingly, the network pharmacological analysis suggests that the most likely mechanism of IO1 activity is through activation of acetylcholine receptors and PTK2 leading to downstream activation of the AKT and MAPK pathways to promote myoblast differentiation. To verify the prediction obtained from the network pharmacology analysis, the promyogenic effects of four constituents (di-*n*-butyl sebacate, 1-monopalmitin, 12-hydroxyoctadecanoic acid, and methyl 3-methoxypropionate) were examined in C2C12 myoblasts (Figure S4). Consistent with the network pharmacology analysis, these compounds exhibited promyogenic effects, as evidenced by the expression of Ki67 (a proliferation marker), Myf5, and MyoD (markers for muscle progenitors), as well as MyoG and Myh3 (differentiation markers) (Figure S4).

### Preventive effects of IO (IO1) on DEX-induced myotube atrophy through AKT activation

**Figure 3:**
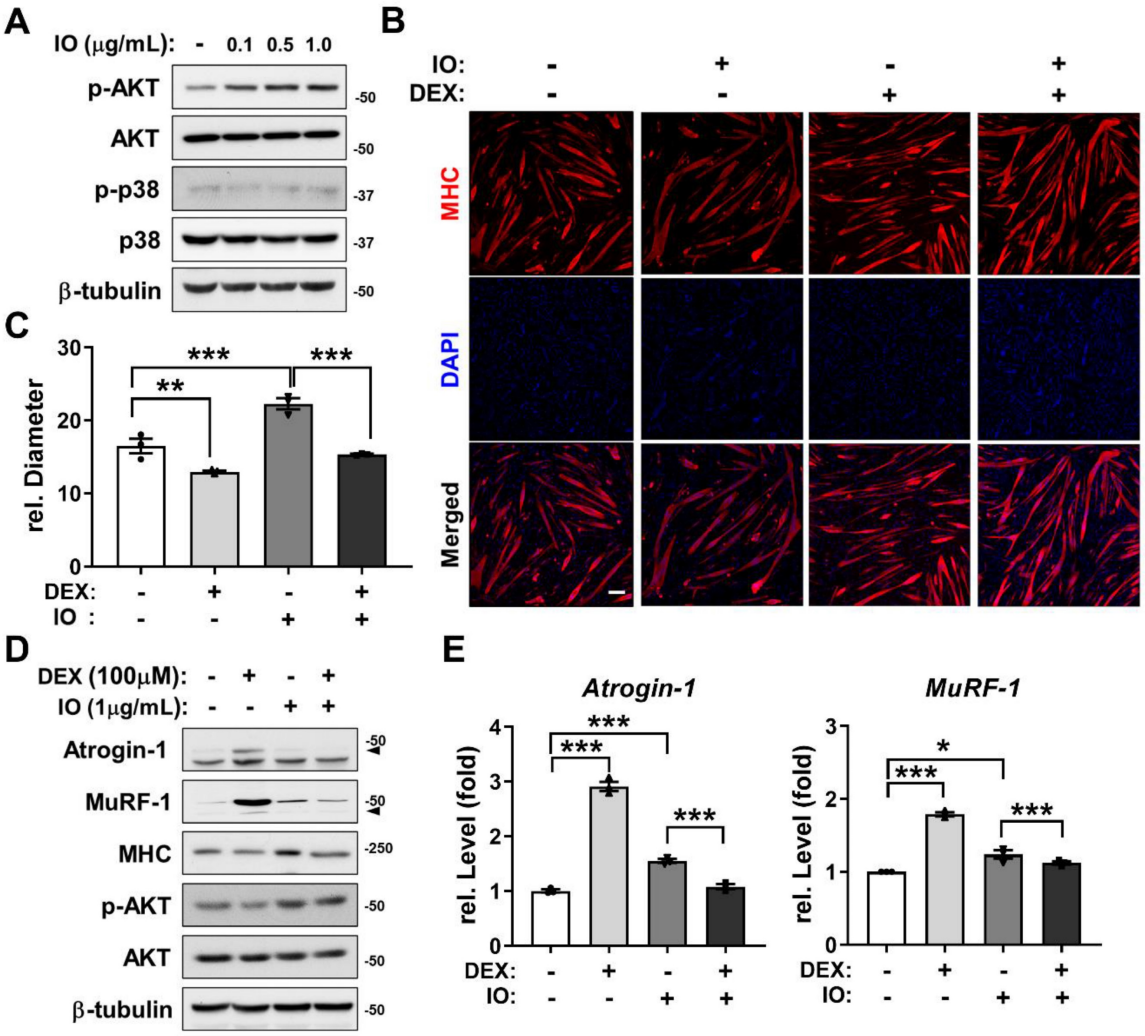
** Effect of IO on destruction of DEX-induced muscle atrophy model through AKT activation.** (A) C2C12 cells were differentiated in DM for 1 day and treated with indicated concentration of IO. Cell lysates were subjected to immunoblotting with antibodies against p-AKT, AKT, p-p38, p38, and β-tubulin. (B) Immunostaining for MHC expression in C2C12 cells treated with normal DM for 3 days and treated with vehicle, IO (1.0 µg/mL), DEX (100 µM), or both IO and DEX for 1 day in DM. Scale bar, 50 µm. (C) Quantification of MHC-positive myotube diameter per field, *n* = 3. (D) C2C12 cells were differentiated in DM for 3 days and treated with vehicle, IO (1.0 µg/mL), DEX (100 µM), or both IO and DEX for 1 day in DM. Cell lysates were subjected to immunoblotting with antibodies against Atrogin-1, MuRF-1, MHC, p-AKT, AKT, and β-tubulin. (E) Expression of Atrogin-1 and MuRF-1 in C2C12 cells treated with normal DM for 3 days and treated with vehicle, IO (1.0 µg/mL), DEX (100 µM), or both IO and DEX for 1 day in DM. The data were normalized using 18s RNA. Atrogin-1 and MuRF-1 expression levels were further normalized to the expression level of vehicle (DMSO), *n* = 3. Data are expressed as the means ± SD. To determine statistical significance, an unpaired two-tailed Student's *t*-test was used. *P < 0.05, **P < 0.01 and ***P < 0.001.

AKT functions as a promyogenic kinase in myoblast differentiation and as an essential regulator of muscle protein synthesis and hypertrophy 33, 35. To examine whether the promyogenic effect of IO (IO1) is through AKT activation as predicted from the network pharmacological analysis, C2C12 myoblasts were induced to differentiate in DM for 1 day, treated with the indicated concentrations of IO, and the activation status of AKT and p38 was assessed by immunoblotting. The treatment of IO dramatically increased p-AKT levels in a dose-dependent manner without changes in the levels of p-p38 and total form of AKT and p38 (Figure 3A). AKT activation attenuates the induction of muscle-specific ubiquitin ligases triggered by synthetic glucocorticoid DEX thereby preventing muscle atrophy 36. To examine whether IO can protect DEX-induced C2C12 myotube atrophy, C2C12 cells were treated with DEX alongside with vehicle DMSO or IO and subjected to MHC immunostaining to assess the thickness of the myotubes. The DEX-elicited myotube atrophy was suppressed by IO treatment, as evident by the larger multinucleated myotubes in IO-treated cultures (Figure 3B and C). In addition, the IO treatment abrogated the DEX-induced elevation of protein levels of muscle-specific E3 ligases, Atrogin-1 and MuRF-1 (Figure 3D). The rescue effect of IO treatment on DEX-induced atrophic myotubes was also reflected by the restored MHC expression close to the level of vehicle control. Furthermore, DEX-treated myotubes exhibited decreased level of p-AKT while IO treatment in DEX-treated myotubes abrogated this decrease. Consistent with the immunoblotting analysis, qRT-PCR analysis showed that DEX treatment greatly elevates the expression of muscle-specific E3 ubiquitin ligases, Atrogin-1 and MuRF-1, while the treatment with IO in DEX-treated myotubes significantly diminishes the mRNA level of Atrogin-1 and MuRF-1 (Figure 3E). Taken together, these results suggest that IO rescues DEX-induced myotube atrophy through inhibition of muscle-specific ubiquitin ligases mediated by Akt activation.

### Effect of IO on skeletal muscle regeneration in CTX-injury mouse model

**Figure 4:**
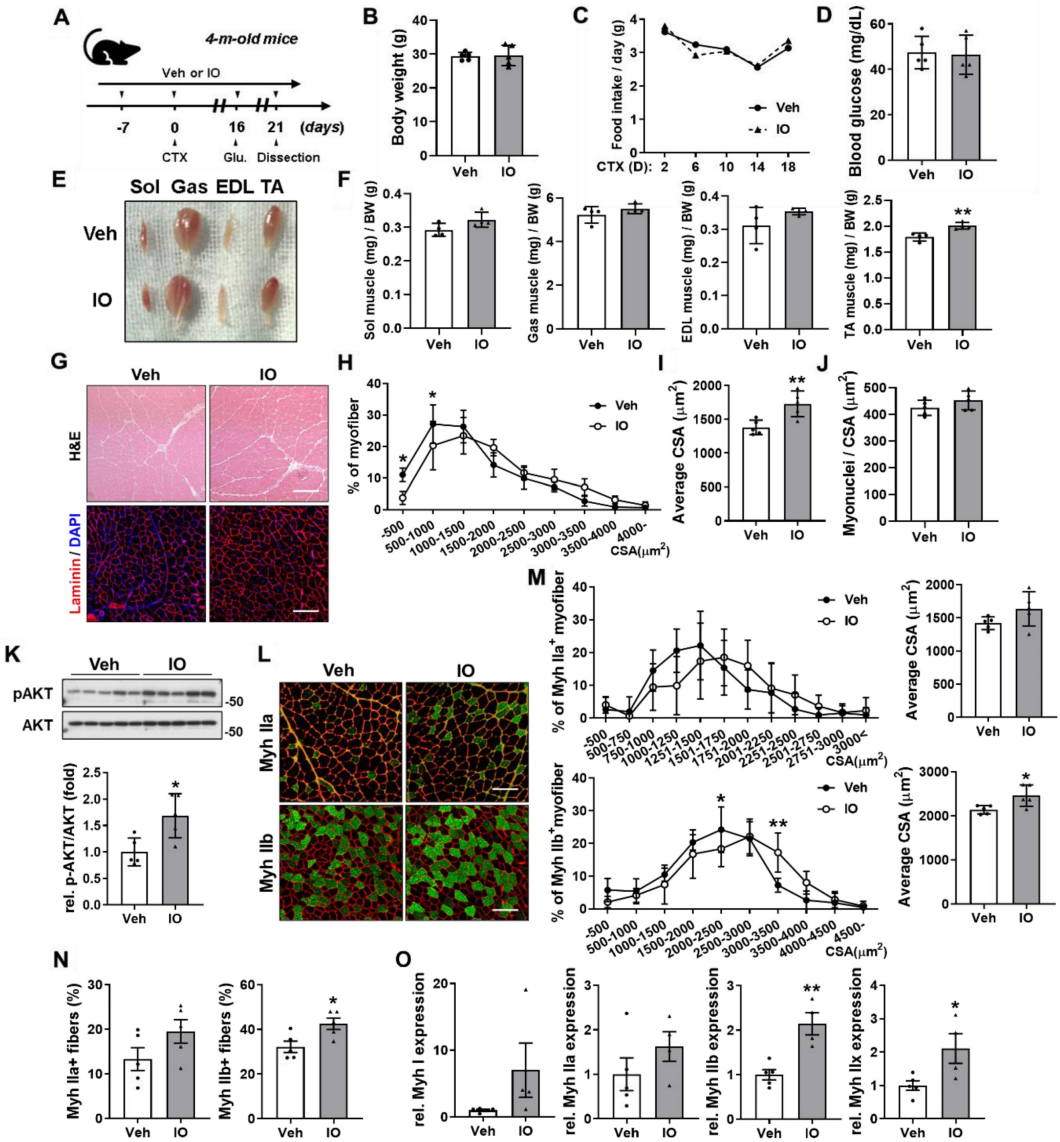
** Effect of IO on skeletal muscle regeneration in *in vivo* mice model at day 21 post-CTX-injection.** (A) Experimental set-up. (B-D) Body weights (B), changes in food intake (C), and blood glucose level (D) of Vehicle- and IO-treated mice, *n* = 5. (E) Photographs of isolated hindlimb muscles from 4-month-old mice ingested with vehicle or 4 mg/kg IO for 4 weeks. (F) Weights of four muscle types from vehicle or 4 mg/kg IO ingested mice for 4 weeks, *n* = 4. (G) Representative H&E and Laminin staining of TA muscle sections from vehicle- and IO-ingested mice. Scale bar, 100 µm. (H) Distribution of the myofibers based on their cross-sectional area (CSA) from panel G, *n* = 5. (I) Quantification of the average myofiber CSA from panel G, *n* = 5. (J) Quantification of myonuclei per CSA from panel G, *n* = 5. (K) Western blot analysis for the expression of p-AKT and AKT in the TA muscles from 4-month-old mice ingested with Vehicle or 4 mg/kg IO for 4 weeks, *n* = 5. (L) Immunostaining of MyhIIa (green), MyhIIb (green), and laminin (red) in the TA muscles of vehicle- or 4 mg/kg IO-ingested 4-month-old mice for 4 weeks. Scale bar, 100 µm. (M) Distribution of MyhIIa- and MyhIIb- positive myofibers based on their CSA and average myofiber CSA from panel K. Quantification of MyhIIa- and MyhIIb-positive myofibers, *n* = 5. (N) Quantification of fiber-type content in TA muscle, *n* = 5. (O) Expression of slow and fast muscle-associated genes in TA muscles. Myh gene expressions by qRT-PCR. MyhI is represented as a slow muscle type, whereas MyhIIa, MyhIIb, and MyhIIX are fast muscle types, *n* = 5 (Veh), *n* = 4 (IO). Data are expressed as the means ± SD. Asterisks indicate significant difference from the control. *P < 0.05 and **P < 0.01.

To further examine the role of IO in muscle regeneration, acute injury was induced by cardiotoxin (CTX) injection in the tibialis anterior (TA) muscle of four-month-old wild type male mice. Starting from a week before CTX injection, mice were orally administered with vehicle or 4 mg/kg IO daily and dissected on day 21 after CTX injection (Figure 4A). IO treatment did not incur changes in body weight, food intake, and blood glucose level compared with the vehicle treatment (Figure 4B-D). The hindlimb muscles of the IO-treated mice appeared darker than those of control mice, which could be readily detected in gastrocnemius (GAS) muscle (Figure 4E). Among four hindlimb muscles, TA muscle displayed the most significant increase in mass per body weight with approximately 12% increase in the IO-fed mice compared to the vehicle-fed mice (Figure 4F). Sections of the TA muscle also revealed an increase in the cross-sectional areas (CSA) of myofibers by IO treatment (Figure 4G-I). In addition, treatment with IO increased the number of myonuclei per CSA in TA muscle fibers compared to the treatment with vehicle (Figure 4J). Next, we examined the effects of IO on the activation of AKT, a key anabolic mediator. IO-treated muscles had significantly enhanced p-AKT expression levels compared with the vehicle muscles (Figure 4K). Taken together, these data suggest that IO treatment improves muscle regeneration in CTX-injured muscles.

To investigate the effect of IO on myofiber types, CTX-injected TA muscle sections were analyzed by immunostaining with antibodies against MyhIIa and MyhIIb. The IO treatment significantly increased the number and CSA of MyhIIb-positive myofibers and increased the number and CSA of MyhIIa-positive myofibers without statistical significance compared with the vehicle treatment (Figure 4L-N). Furthermore, qRT-PCR analysis of TA muscles from vehicle- and IO-fed mice revealed that IO treatment significantly increases the expression of both oxidative myofiber markers, MyhI and MyhIIa, and glycolytic myofiber markers, MyhIIb and MyhIIx (Figure 4O). These data indicate that IO treatment enhances the regeneration of both oxidative and glycolytic myofiber types.

### Effect of IO on muscle stem cell proliferation in regenerating muscles at day 3 post-CTX-injury

**Figure 5:**
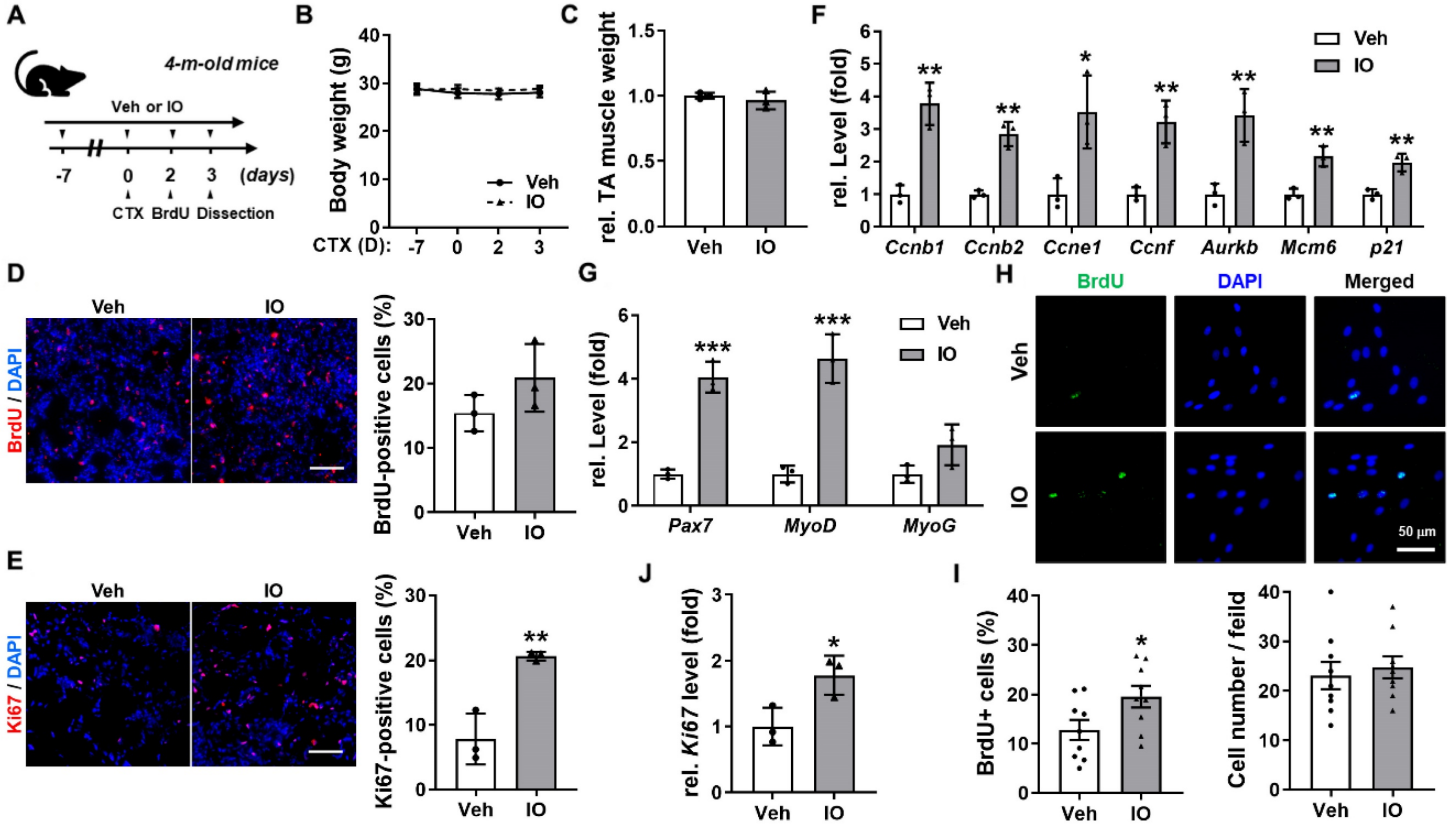
** Effect of IO on muscle stem cell proliferation in *in vivo* mice model at 3 days post-CTX-injection.** (A) The experimental scheme. (B) The change of body weight for total 10 days of the experiment, *n* = 3. (C) The relative TA muscle weight normalized to body weight, *n* = 3. (D) Immunostaining of BrdU incorporation and quantification of BrdU-positive cells in Vehicle- or IO treated TA muscles post 3 days of CTX injury, *n* = 3. (E) Immunstaining for Ki67 and quantification of Ki67-positive cells in TA muscles, *n* = 3. (F & G) Quantitative RT-PCR analysis of cell cycle markers (F) and early myogenic regulatory factors (G) in TA muscles, *n* = 3. The data were normalized to 18s RNA levels and were further normalized to the expression level of vehicle. (H) Immunostaining of BrdU incorporation in Vehicle- or IO-treated aged human myoblasts (66-year-old). (I) Quantification of BrdU-positive cells and cell number per field from data shown in panel H, *n* = 9. (J) Quantitative RT-PCR analysis of Ki67 in Vehicle- or IO-treated aged human myoblasts (66-year-old), *n* = 3. Data are expressed as the means ± SD. Asterisks indicate significant difference from the control. *P < 0.05, **P < 0.01 and ***P < 0.001.

To investigate the effect of IO at the early stage of muscle regeneration, four-month-old wild type male mice were orally administered with either vehicle or 4 mg/kg IO daily starting from 7 days prior to CTX injection in TA muscles and the muscles were harvested post 3 days of injury (Figure 5A). The IO treatment did not induce changes in body weight and relative TA muscle weight compared with the vehicle treatment (Figure 5B and C). To monitor the proliferation in the regenerating muscles, cryosections of TA muscles were subjected to immunostaining for BrdU incorporation and Ki67. IO treatment elevated the number of BrdU and Ki67-positive cells compared to the vehicle treatment (Figure 5D and E). Furthermore, IO treatment significantly increased the expression levels of genes expressed during cell proliferation (Ccnb1, Ccnb2, Ccne1, Ccnf, Aurkb, Mcm6, and p21) and markers for activated proliferating muscle stem cells (Pax7 and MyoD), suggesting that the improved muscle regeneration by IO treatment is attributable to the increased proliferation of muscle stem cells (Figure 5F and G). Aged human myoblasts treated with IO also exhibited increased proliferation compared with the vehicle-treated cells, further verifying the effect of IO on the muscle stem cell proliferation (Figure 5H and I). In parallel with Figure 5H & I, IO treatment significantly increased the Ki67 expression (Figure 5J). These results suggest that IO enhances the activation and proliferation of muscle stem cells in response to muscle injury.

### Effect of IO on the oxidative muscle metabolism

**Figure 6:**
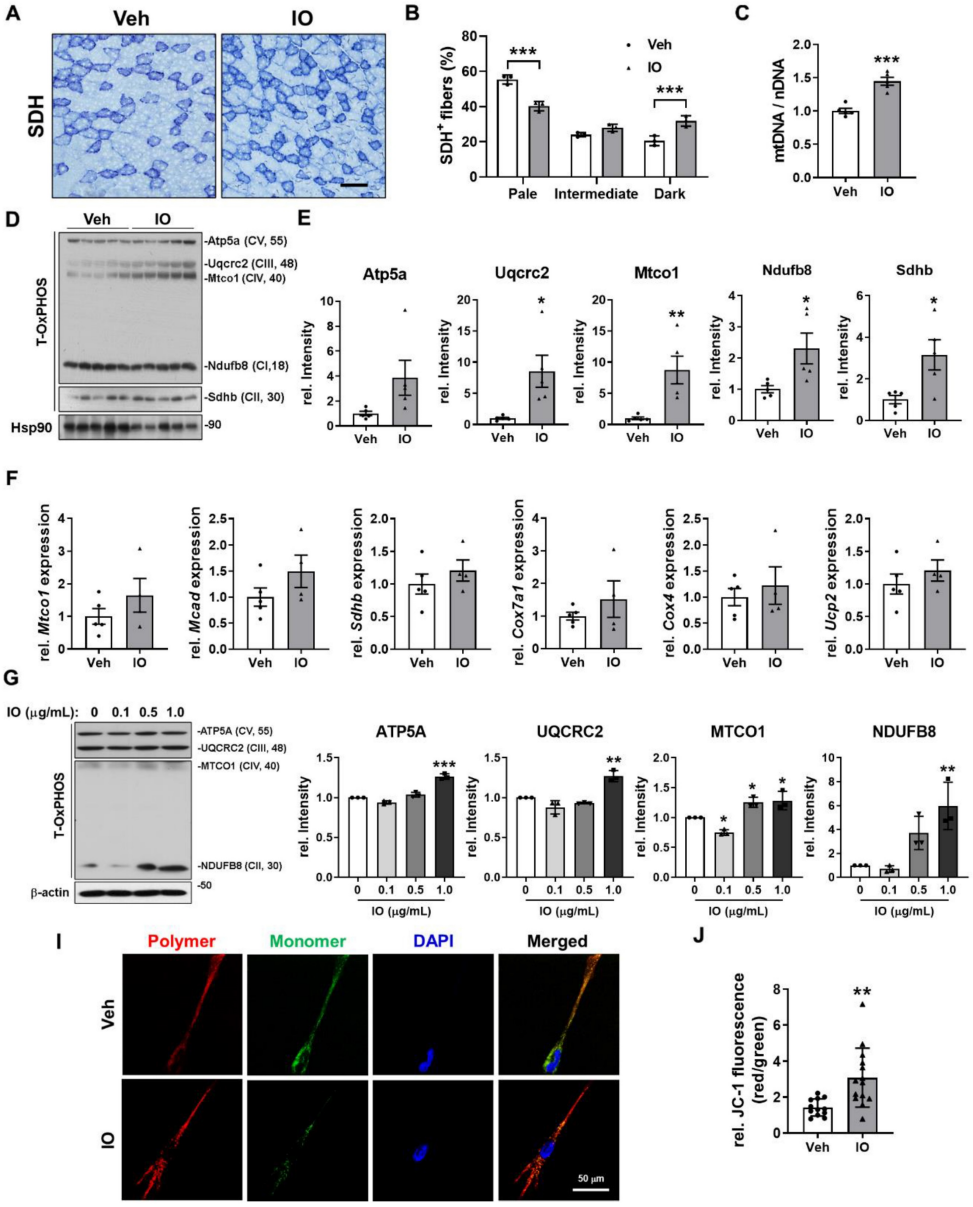
** Effect of IO on the oxidative muscle metabolism.** (A) Histochemical staining for SDH enzymatic activities in Vehicle- or IO-treated TA muscle. Scale bar, 100 µm. (B) The staining intensities of SDH are quantified as three different grades (dark, intermediate, and pale) and plotted as a percentile, *n* = 3. (C) Relative mitochondrial DNA content in TA muscles from 4-month-old mice ingested with Vehicle or 4 mg/kg IO for 4 weeks, *n* = 5. (D) Western blot analysis for the expression of total-OxPHOS in TA muscles from 4-month-old mice ingested with Vehicle or 4 mg/kg IO for 4 weeks. (E) Quantification of the relative levels of total-OxPHOS proteins from panel D, *n* = 5. (F) The expression of genes involved in the regulation of mitochondrial function examined by qRT-PCR analysis. The data were normalized using 18s RNA and were further normalized to the expression level of vehicle, *n* = 5 (Veh), 4 (IO). (G) Western blot analysis for the expression of total-OxPHOS in C2C12 cells treated with indicated concentration of IO. (H) Quantification of the relative levels of total-OxPHOS proteins from panel G, *n* = 3. (I) JC-1 fluorescence in C2C12 cells treated with either Vehicle or IO1. (J) Quantification of the relative levels of JC-1 from panel I. Data are expressed as the means ± SD. To determine statistical significance, an unpaired two-tailed Student's *t*-test was used. *P < 0.05, **P < 0.01 and ***P < 0.001.

Since IO-treated mice exhibited darker hindlimb muscles and substantially increased expression of MyhI and MyhIIa compared with the vehicle-treated mice (Figure 4E and N), we presumed that IO may also have an effect in promoting oxidative muscle metabolism. TA muscles of 4-month-old mice treated with either vehicle or 4 mg/kg of IO for 4 weeks were subjected to histochemical staining for the activity of mitochondrial enzyme succinate dehydrogenase (SDH). IO treatment elevated the proportion of myofibers with strong (dark) and intermediate staining for SDH compared to the vehicle treatment (Figure 6A and B). In addition, IO significantly enhanced the expression of total OxPHOS (Atp5a, Uqcr2, Mtco1, NdufB8, and Sdhd) and mitochondrial genes (Mtco1, Mcad, Sdhd, Cox7a1, Cox4, and Ucp2) (Figure 6C-E). The relative mitochondrial DNA content was also significantly increased by IO treatment, indicating that IO enhances oxidative muscle metabolism (Figure 6F). To confirm these effects of IO, C2C12 myoblasts were induced to differentiate with the indicated concentrations of IO in DM and subjected to immunoblotting analysis. In agreement with the results from the mice model, IO treatment significantly increased the expression of total OxPHOS and mitochondrial genes in a dose dependent manner (Figure 6G and H). Finally, IO-treated C2C12 cells exhibited increased mitochondrial membrane potential as evidenced by the increased JC-1 polymer/monomer ratio compared with the vehicle-treated cells (Figure 6I and J). These results suggest that IO enhances the oxidative muscle metabolism through upregulation of mitochondrial gene expression.

### Effect of IO on the expression of PGC-1α in young mice skeletal muscle and C2C12 cells

**Figure 7:**
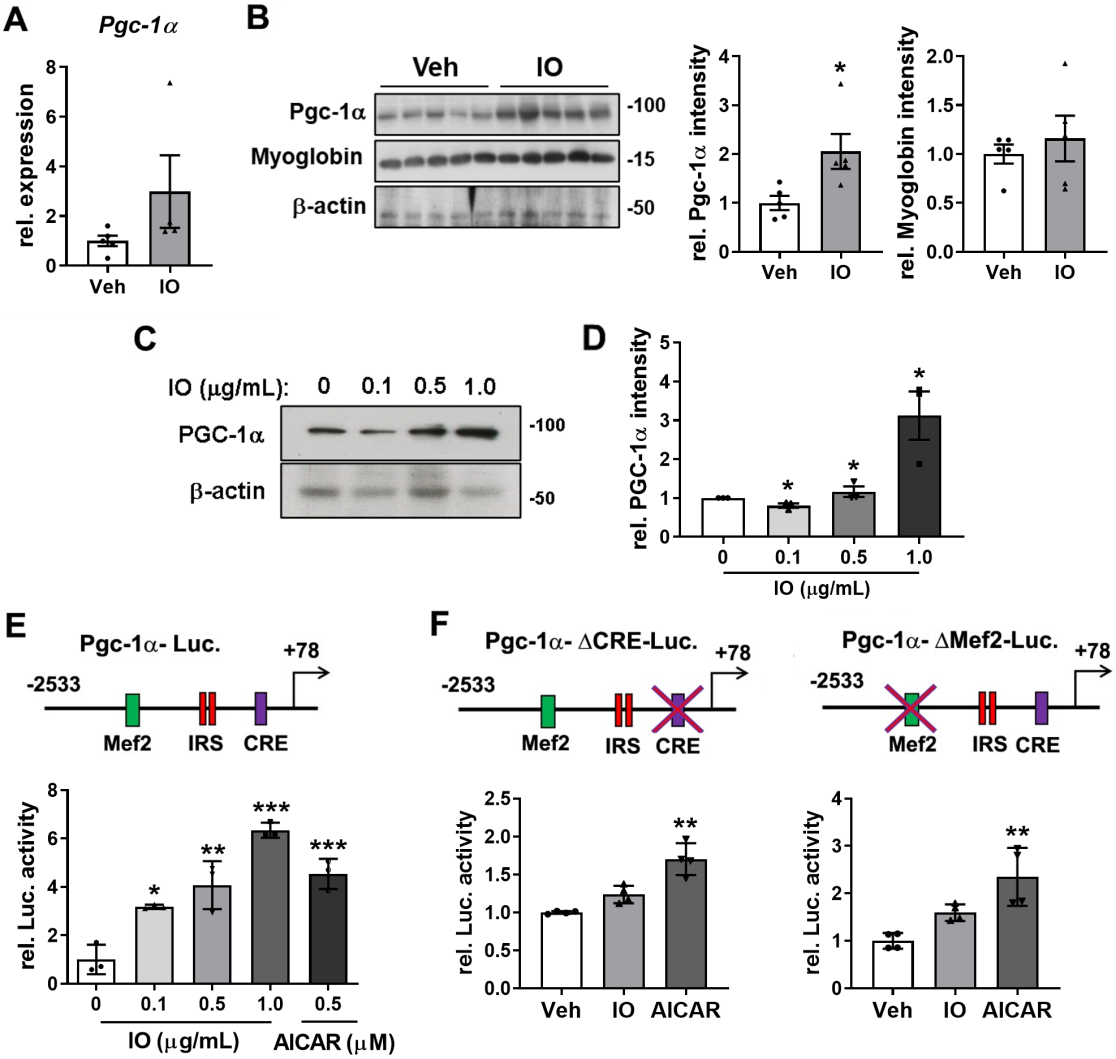
** Effect of IO on the expression of PGC-1α in young mice skeletal muscle and C2C12 myoblasts.** (A) Relative expression of PGC-1α in TA muscles from 4-month-old mice ingested with Vehicle or 4 mg/kg IO for 4 weeks, *n* = 5 (Veh), 4 (IO). (B) Western blot analysis and quantification of the relative levels of PGC-1α and myoglobin proteins in TA muscles from 4-month-old mice ingested with Vehicle or 4 mg/kg IO for 4 weeks, *n* = 5. (C) Western blot analysis for the expression of PGC-1α in C2C12 cells treated with indicated concentration of IO. And (D) quantification of the relative levels of PGC-1α protein, *n* = 3. (E) The relative PGC-1α luciferase activity in C2C12 cells treated with vehicle (DMSO) or indicated concentration of IO or AICAR as a positive control, *n* = 3. (F) The relative PGC-1α luciferase activity in C2C12 cells treated with vehicle (DMSO), IO (1.0 µg/mL), or AICAR (0.5 mM), *n* = 4. Data are expressed as the means ± SD. To determine statistical significance, an unpaired two-tailed Student's *t*-test was used. Asterisk indicates statistical significance. *P < 0.05, **P < 0.01 and ***P < 0.001 (IO vs. Vehicle).

PGC-1α is a transcriptional coactivator that activates the expression of genes involved in mitochondrial biogenesis, stimulation of fatty acid oxidation, and resistance to muscle atrophy 12. Thus, we investigated whether IO regulates the oxidative muscle metabolism through regulation of PGC-1α expression. The mRNA and protein levels of PGC-1α and myoglobin were elevated in the IO-treated TA muscles compared with the vehicle-treated muscles (Figure 7A and B). In agreement with the data of TA muscles, IO treatment increased the level of PGC-1α mRNA as well as protein up to approximately 3.0-fold in C2C12 myoblasts (Figure 7C and D). To further define the mechanism of IO in the activation of PGC-1α expression, the reporter activity of full-length or mutant PGC-1α promoters (with a deletion of either MEF2 or CRE motif) was measured in C2C12 cells. The full-length promoter-driven luciferase activity was significantly upregulated by IO1 in a dose-dependent manner, with 1.0 µg/mL IO treatment resulting in higher luciferase activity than the treatment with 0.5 mM (1.313 mg/mL) AICAR, which is a known activator of PGC-1α through AMPK activation (Figure 7E). The deletion of MEF2 or CRE motif abrogated the reporter activity upregulation by IO to a greater degree than and it did to the reporter activity upregulation by AICAR (Figure 7F), suggesting for the requirement of both elements to mediate the effect of IO on PGC-1α expression. Taken together, these data suggest that IO upregulates the expression of PGC-1α to promote oxidative muscle metabolism.

## Discussion

*I. obliquus* has been reported to have various health promoting properties which include antitumor, antioxidant, antiviral, anti-inflammatory, and immunomodulatory activity without prominent side effects (reviewed in 37,38). However, most studies were performed in the context of cancer cells with a few exceptions of normal cell lines from digestive system such as pancreatic and hepatic cell lines. There have been limited investigation to our knowledge on its effect on myogenesis and regeneration of skeletal muscle. In this study, we investigated the effect of IO treatment on muscle health and the potential mechanism underlying the effect. IO treatment promoted C2C12 myoblast differentiation and alleviated DEX-induced myotube atrophy. IO treatment also improved muscle regeneration of CTX-injury mouse accompanied by an increase in muscle stem cell proliferation and oxidative muscle metabolism. Network pharmacological analysis of IO predicted that the promyogenic function of IO is mediated by the activation of AKT pathway, which was confirmed in C2C12 myoblasts. Taken together, the data here present a positive effect of IO on myogenesis and muscle regeneration in both *in vivo* and *in vitro* model of muscle atrophy through AKT-dependent mechanisms.

Investigation on the bioactive ingredients of IO and the molecular mediators of its biological effect is yet minimal and still ongoing. By comparing the metabolite profiles of IO1 and IO2, we identified 6 chemical constituents of IO as the potential bioactive compounds responsible for the myogenic effect of IO (Figure 2C; Table S1). Based on the network pharmacological analysis using the identified 6 chemical constituents of IO, we propose two major mediators of IO in the skeletal muscle: AKT and PGC-1α. Previous studies have shown the activation of PI3K/AKT signaling pathway by treatment of IO or its component in other contexts 39,40. In both *in vivo* and *in vitro* muscle models, we also observed an increase in phosphorylated AKT in response to IO. We present here the AKT activation as one of the potential mechanisms underlying the rescue effect of IO on muscle atrophy. Currently, how IO regulates the crosstalk between AKT/muscle hypertrophy and PGC-1α/mitochondria pathways is unclear. It is plausible that AKT activation might induce AMPK/PGC-1α signaling as feedback, leading to mitochondrial biogenesis.

A similar mechanism has been observed in resistance exercise-mediated enhancement in muscle mass and strength 41. A recent study has also reported that muscle Akt is required for the maintenance of oxidative muscle metabolism and protein synthesis to enhance muscle mass and strength 42. Akt signaling also prevents immobilization-induced muscle wasting 43. Conversely, the inhibition of Akt signaling in muscle causes a shift towards non-oxidative muscle and mitochondrial dysfunction, with the concomitant loss of exercise performance 42,43. AKT-deficient skeletal muscles display a substantial reduction in Pgc1α/β expression, resulting in the loss of mitochondrial function 42. Thus, it is likely that IO might regulate both AKT/muscle hypertrophy and PGC1α/mitochondria via AKT signaling. Further studies are needed to define the exact molecular mechanism.

PGC-1α is a key regulator of muscle metabolism via modulating mitochondria biosynthesis 44. Of the 6 potential bioactive compounds of IO, 10-oxo-*cis*-12-octadecenoic acid and 12-hydroxyoctadecanoic acid were shown to strongly activate peroxisome proliferator-activated receptors (PPARs), which in turn regulate the activity and expression of PGC-1α 45,46. The expression of PGC-1α was indeed significantly increased by IO treatment in both mice and C2C12 myoblast (Figure 7A-D). Moreover, increased oxidative muscle metabolism by IO treatment was indicated by the increase in SDH enzymatic activity, expression of total OxPHOS and mitochondrial genes, and relative mitochondrial DNA content in TA muscles of IO-treated mice (Figure 6A-F). Since mitochondrial function is important during regeneration for energy production, homeostasis of reactive oxygen species, cross talk with immune cells, and modulation of stem cell fate, the enhanced mitochondrial function by IO through PGC-1α may also contribute to the improved muscle regeneration.

Taken together, our study demonstrates a promyogenic effect of IO in the context of muscle regeneration following injury through modulation of AKT signaling pathway and oxidative muscle metabolism. As imbalance in protein homeostasis and mitochondrial dysfunction are the key characteristics of aged and diseased muscle, IO is a promising drug candidate to promote muscle health.

## Conclusions

IO treatment resulted in the activation of AKT, which inhibits the muscle-specific ubiquitin E3 ligase induced by dexamethasone. IO treatment showed improved regeneration of muscle with increased proliferation and differentiation of muscle stem cells and increased mitochondrial content and muscle oxidative metabolism with induction of PGC-1α. These results suggest that IO is considered a promising drug candidate for promoting muscle health with promyogenic effects that overcome the imbalance of protein homeostasis and mitochondrial dysfunction seen in aging through modulation of the AKT signaling pathway and oxidative muscle metabolism.

## Supplementary Material

Supplementary figures and table.

## Figures and Tables

**Table 1:** The primers used in this study

Primer		Sequence
eMHC	Forward	5'-CTGGAGTTTGAGCTGGAAGG-3'
	Reverse	5'-CAGCCTGCCTCTTGTAGGAC-3'
Myogenin	Forward	5'-ATCTCCGCTACAGAGGCGGG-3'
	Reverse	5'-TAGGGTCAGCCGCGAGCAAA-3'
Atrogin-1	Forward	5'-CAACATTAACATGTGGGTGTAT-3'
	Reverse	5'-GTCACTCAGCCTCTGCATG-3'
MuRF1	Forward	5'-GAGAACCTGGAGAAGCAGCT-3'
	Reverse	5'-CCGCGGTTGGTCCAGTAG-3'
MyhI	Forward	5'-ACAAGCTGCAGCTGAAGGTG-3'
	Reverse	5'-TCATTCAGGCCCTTGGCAC-3'
MyhIIa	Forward	5'-CCAGCTGCACCTTCTCGTTTGCCAG-3'
	Reverse	5'-CATGGGGAAGATCTGGTCTTCTT-3'
MyhIIb	Forward	5'-CCTGGAACAGACAGAGAGGAGCAGGAGAG-3'
	Reverse	5'-GTGAGTTCCTTCACTCTGCGCTCGTGC-3'
MyhIIX	Forward	5'-TGCAACAGTTCTTCAACCAC-3'
	Reverse	5'-GCCAGGTCCATCCCAAAGT-3'
Myh3	Forward	5'-CTTCACCTCTAGCCGGATGGT-3'
	Reverse	5'-AATTGTCAGGAGCCACGAAAAT-3'
Pgc-1α	Forward	5'-ATGTGTCGCCTTCTTGCTCT-3'
	Reverse	5'-CGGTGTCTGTAGTGGCTTGA-3'
Mtco1	Forward	5'-CTACTATTCGGAGCCTGAGC-3'
	Reverse	5'-GCATGGGCAGTTACGATAAC-3'
Mcad	Forward	5'-GGTTTGGCTTTTGGACAATG-3'
	Reverse	5'-TGACGTGTCCAATCTACCACA-3'
Sdhb	Forward	5'-CAGAGTCGGCCTGCAGTTTC-3'
	Reverse	5'-GGTCCCATCGGTAAATGGCA-3'
Cox7a1	Forward	5'-GTCTCCCAGGCTCTGGTCCG-3'
	Reverse	5'-CTGTACAGGACGTTGTCCATTC-3'
Cox4	Forward	5'-CTATGTGTATGGCCCCATCC-3'
	Reverse	5'-AGCGGGCTCTCACTTCTTC-3'
Ucp2	Forward	5'-ACTGTCGAAGCCTACAAGAC-3'
	Reverse	5'-CACCAGCTCAGTACAGTTGA-3'
Tnfα	Forward	5'-AGCCCCCAGTCTGTATCCTT-3'
	Reverse	5'-CTCCCTTTGCAGAACTCAGG-3'
Il6	Forward	5'-GGTGACAACCACGGCCTTCCC-3'
	Reverse	5'-AAGCCTCCGACTTGTGAAGTGGT-3'
Il10	Forward	5'-GCCAAGCCTTATCGGAAATG-3'
	Reverse	5'-CACCCAGGGAATTCAAATGC-3'
Il1RA	Forward	5'-TTCTTGTTGCCTCTGCCACTCG-3'
	Reverse	5'-GATTGGTCTGGACTGTGGAAGTG-3'
Ccl5	Forward	5'-TGCCCACGTCAAGGAGTATTT-3'
	Reverse	5'-TTCTCTGGGTTGGCACACACT-3'
Ccl22	Forward	5'-AAGACAGTATCTGCTGCCAGG-3'
	Reverse	5'-GATCGGCACAGATATCTCGG-3'
Cxcl1	Forward	5'-TGAGCTGCGCTGTCAGTGCC-3'
	Reverse	5'-AGAAGCCAGCGTTCACCAGA-3'
Pax7	Forward	5'-GAGTTCGATTAGCCGAGTGC-3'
	Reverse	5'-CGGGTTCTGATTCCACATCT-3'
MyoD	Forward	5'-GATGGCATGATGGATTACAGCGGC-3'
	Reverse	5'-GTGGAGATGCGCTCCACTATGCTG-3'
Ki67 (mouse)	Forward	5'-CTGCCTCAGATGGCTCAAAGA-3'
	Reverse	5'-GAAGACTTCGGTTCCCTGTAAC-3'
Ki67 (human)	Forward	5'-TCCCGCCTGTTTTCTTTCTGAC-3'
	Reverse	5'-CTCTCCAAGGATGATGATGCTTTAC-3'
Myf5	Forward	5'-CTCAGGAATGCCATCCGCTACATTGAGA-3'
	Reverse	5'-ATCCAAGCTGGATAAGGAGCTTTTATCCG-3'
18S rRNA (mouse)	Forward	5'-AGGGGAGAGCGGGTAAGAGA-3'
	Reverse	5'-GGACAGGACTAGGCGGAACA-3'
18S rRNA (human)	Forward	5'-GTAACCCGTTGAACCCCATT-3'
	Reverse	5'-CCATCCAATCGGTAGTAGCG-3'

## Abbreviations

AICAR: aminoimidazole carboxamide ribonucleotide
AKT: protein kinase B
Atp5a: ATP synthase subunit alpha
Atrogin-1: F-box only protein 32
Ccl22: c-c motif chemokine ligand 22
Ccl5: c-c motif chemokine ligand 5
Cox4: cytochrome c oxidase subunit 4
Cox7a1: cytochrome c oxidase subunit 7a1
CSA: cross section area
Cxcl1: c-x-c motif chemokine ligand 1
DEX: dexamethasone
DMSO: dimethyl sulfoxide
EDL: extensor digitorum longus
GAS: gastrocnemius
H&E: hematoxylin and eosin
Hsp90: heat shock protein 90
Il10: interleukin-10
Il1Ra: interleukin-1 receptor antagonist
Il6: interleukin-6
Mcad: medium-chain acyl-coenzyme a dehydrogenase
MHC: myosin heavy chain
Mtco1: mitochondrially encoded cytochrome c Oxidase 1
MuRF-1: E3 ubiquitin-protein ligase TRIM63
Myh: myosin heavy chain
MyoD: Myoblast determination protein 1
MyoG: myogenin
NdufB8: NADH dehydrogenase ubiquinone 1 beta subcomplex subunit 8
p38MAPK: p38 mitogen-activated protein kinases
Pax7: paired box 7
Pgc-1α: peroxisome proliferator-activated receptor γ coactivator α
SDH: succinate dehydrogenase
Sdhb: succinate dehydrogenase complex iron sulfur subunit B
SOL: soleus
TA: tibialis anterior
Tnfα: tumor necrosis factor-alpha
Upc2: mitochondrial uncoupling protein 2
