# Supplementary material for: Inonotus obliquus upregulates muscle regeneration and augments function through muscle oxidative metabolism
Source: Int J Biol Sci. 2023 Sep 11;19(15):4898–914. doi: 10.7150/ijbs.84970 (PMC10539711; doi:10.7150/ijbs.84970)

Inonotus obliquus upregulates muscle regeneration and augments function through muscle oxidative metabolism

Chang-Lim You[1#], Sang-Jin Lee[2#], Jinwoo Lee[2], Tuan Anh Vuong[2], Hye-Young Lee[2], Se Yun Jeong[3], Akida Alishir[3], Allison S. Walker[4,5], Gyu-Un Bae[2,6]*, Ki Hyun Kim[3]*, Jong-Sun Kang[1]*

1. Department of Molecular Cell Biology, Sungkyunkwan University School of Medicine, Suwon 16419, Republic of Korea
2. Research Institute of Aging Related Disease, AniMusCure Inc., Suwon 16419, Republic of Korea
3. School of Pharmacy, Sungkyunkwan University, Suwon 16419, Republic of Korea
4. Department of Chemistry, Vanderbilt University, Nashville, TN 37235, United States
5. Department of Biological Sciences, Vanderbilt University, Nashville, TN 37235, United State
6. Drug Information Research Institute, Muscle Physiome Research Center, College of Pharmacy, Sookmyung Women's University, Seoul 04310, Republic of Korea

---

[#]These authors contributed equally to this work

* Corresponding authors:

Jong-Sun Kang, Ph.D., Department of Molecular Cell Biology, Single Cell Network Research Center, Sungkyunkwan University School of Medicine, Suwon 16419, Republic of Korea; Tel.: +82-31-299-6135, Fax.: +82-31-299-6157, E-mail: kangj01@skku.edu

Ki Hyun Kim, Ph.D., School of Pharmacy, Sungkyunkwan University, Suwon 16419, Republic of Korea; Tel: +82-31-290-7700; Fax: +82-31-290-7730; E-mail: khkim83@skku.edu

Gyu-Un Bae, Ph.D., Research Institute of Aging Related Disease, AniMusCure Inc., Suwon 16419, Republic of Korea; Drug Information Research Institute, Muscle Physiome Research Center, College of Pharmacy, Sookmyung Women's University, Seoul 04310, Republic of Korea; Tel.: +82-2-2077-7629, Fax.: +82-2-2077-7629, E-mail: gbae@sookmyung.ac.kr

Table of Contents

Table S1. Characterization of the specific metabolites of IO1 by UPLC-Q-TOF-HRMS.

Figure S1. Promyogenic effects on C2C12 cells in the presence of higher concentration of IO.

Figure S2. Cell viability of C2C12 cells in the presence of high concentration of IO for 1 day.

Figure S3. UPLC Q-TOF MS analyses conducted on IO1 and IO2. The total ion chromatograms (TICs) in positive-ion mode (A) and negative-ion mode (B) of IO1 and IO2 are displayed.

Figure S4. The effects of four selected compounds on the expression of genes involved in proliferation (A) and differentiation (B) using qRT-PCR analysis. The data were normalized using 18s RNA and were further normalized to the expression level of vehicle.

Table S1. Characterization of the specific metabolites of IO1 by UPLC-Q-TOF-HRMS.

Ion mode	Identified compounds	Retention time (min)	Formula	Theoretical (*m/z*)	Experimental (*m/z*)	Adducts / Deprotonated one	Δ (ppm)
Positive-ion mode	10-oxo-*cis*-12-octadecenoic acid	8.606	$C_{18}H_{32}O_3$	319.2249	319.2245	$[M+Na]^+$	2.29
	di-n-butyl sebacate[a]	7.401	$C_{18}H_{34}O_4$	337.2355	337.2350	$[M+Na]^+$	0.15
	1-monopalmitin[a]	9.598	$C_{19}H_{38}O_4$	353.2268	353.2663	$[M+Na]^+$	0.41
	10-oxo-*trans*-8-decenoic acid	5.536	$C_{10}H_{16}O_3$	207.0997	207.0974	$[M+Na]^+$	-1.06
Negative-ion mode	methyl 3-methoxypropanoate[a]	1.218	$C_5H_{10}O_3$	117.0552	117.0559	$[M-H]^-$	0.78
	12-hydroxyoctadecanoic acid[a]	9.692	$C_{18}H_{36}O_3$	299.2586	299.2593	$[M-H]^-$	-0.13

[a] Confirmed by comparison to authentic standards.

Figure S1. Promyogenic effects on C2C12 cells in the presence of higher concentration of IO.

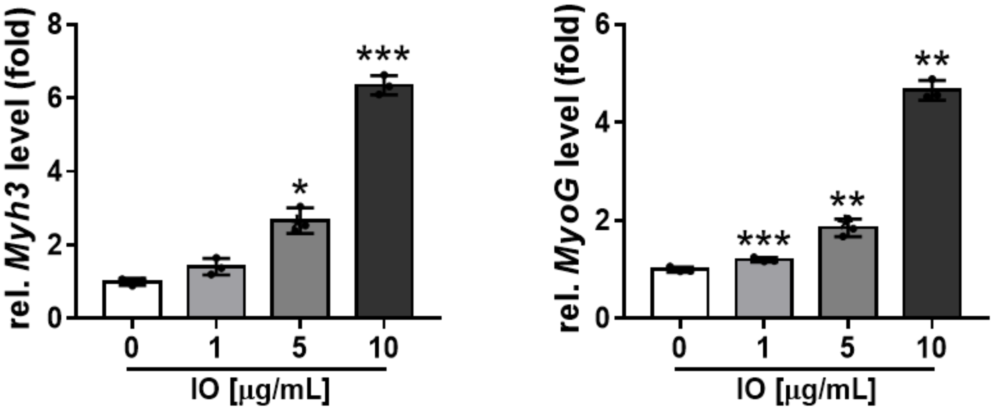

Figure S2. Cell viability of C2C12 cells in the presence of high concentration of IO for 1 day.

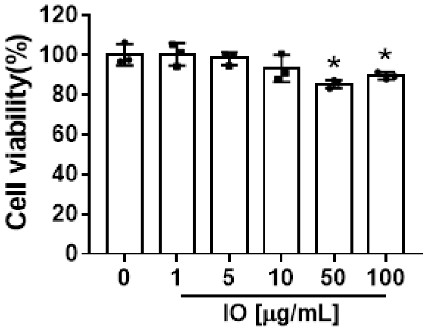

Figure S3. UPLC Q-TOF MS analyses conducted on IO1 and IO2. The total ion chromatograms (TICs) in positive-ion mode (A) and negative-ion mode (B) of IO1 and IO2 are displayed.

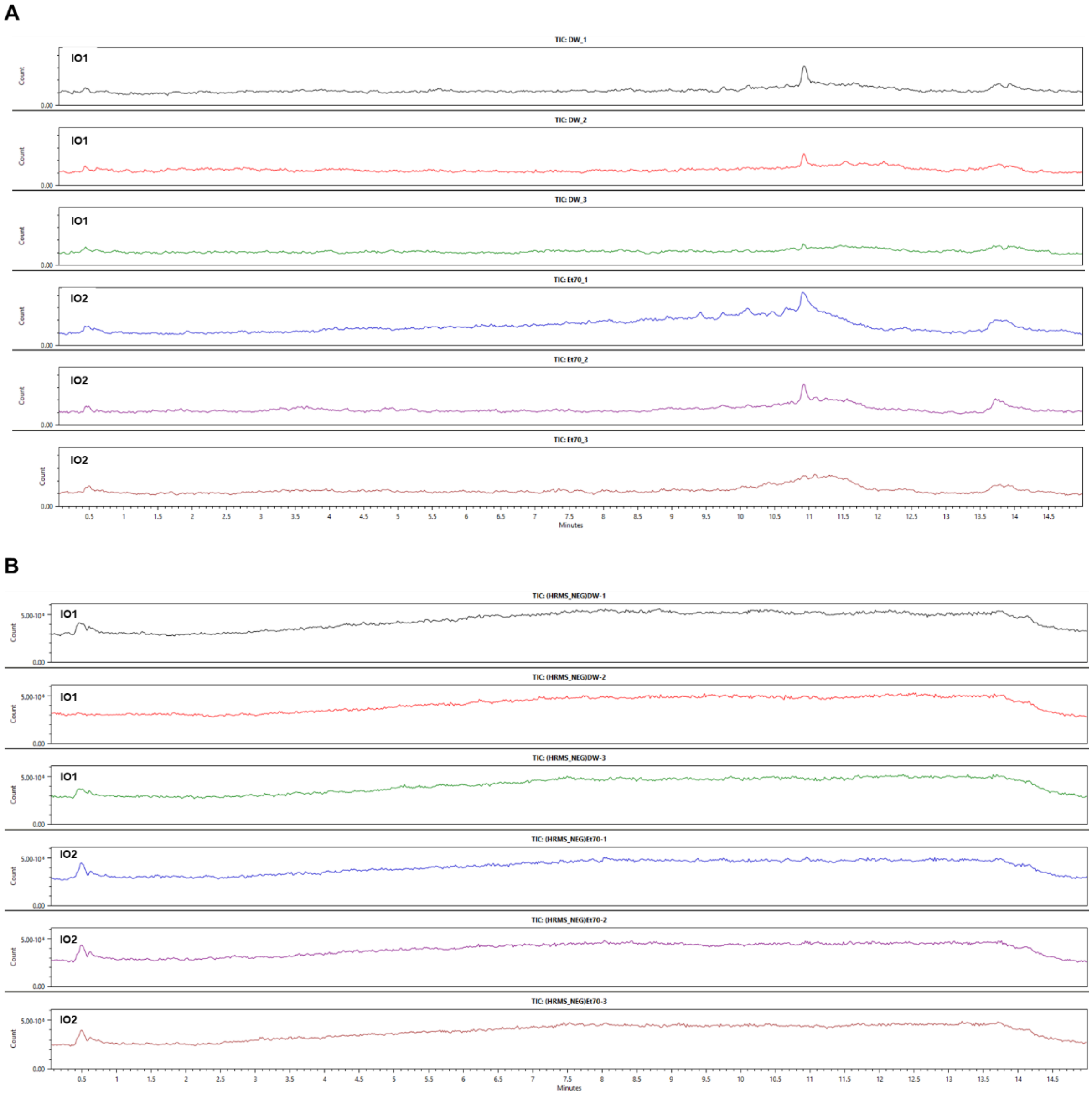

Figure S4. The effects of four selected compounds on the expression of genes involved in proliferation (A) and differentiation (B) using qRT-PCR analysis. The data were normalized using 18s RNA and were further normalized to the expression level of vehicle.

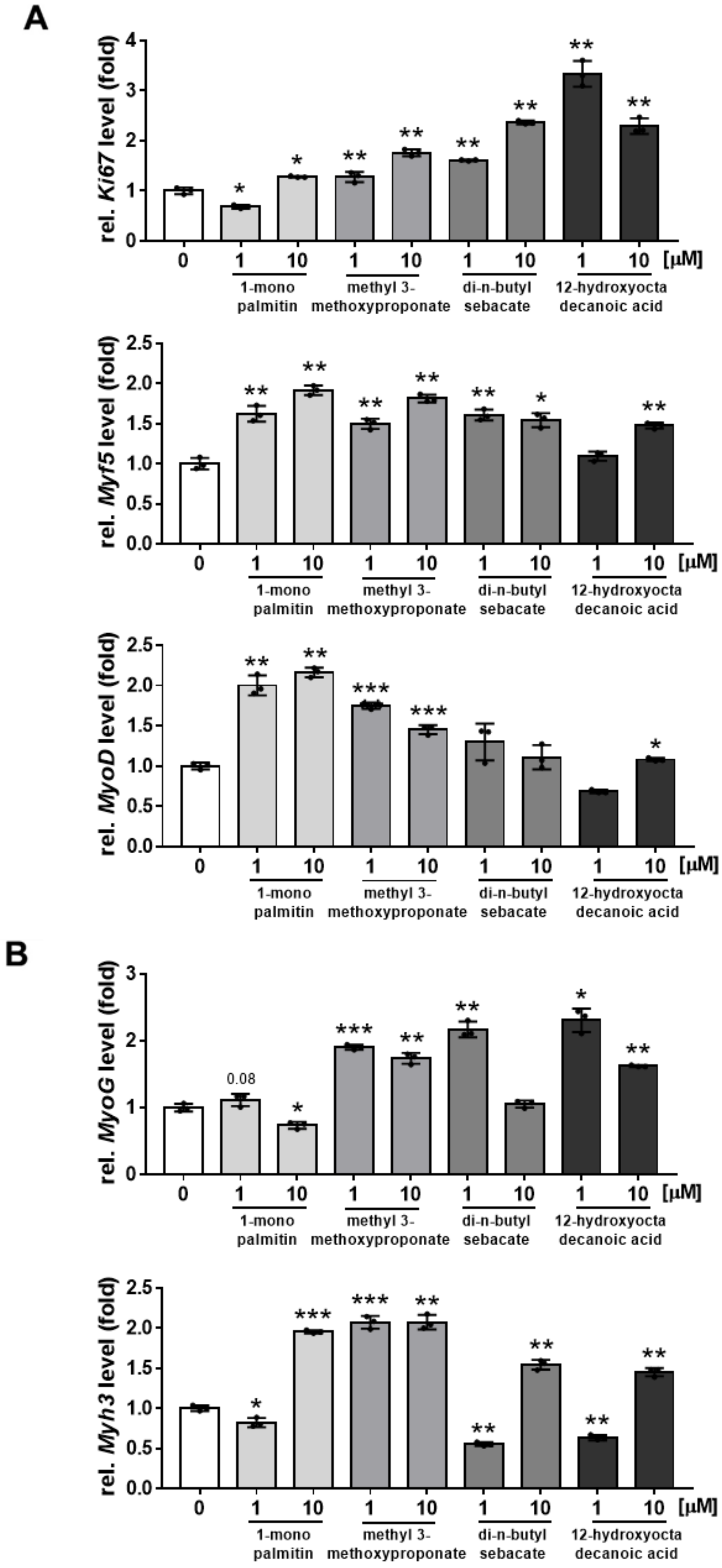

Supplement: Supplementary file 1 — Supplementary figures and table. [file ijbsv19p4898s1.pdf]
